# Does Physical Activity Predict Obesity—A Machine Learning and Statistical Method-Based Analysis

**DOI:** 10.3390/ijerph18083966

**Published:** 2021-04-09

**Authors:** Xiaolu Cheng, Shuo-yu Lin, Jin Liu, Shiyong Liu, Jun Zhang, Peng Nie, Bernard F. Fuemmeler, Youfa Wang, Hong Xue

**Affiliations:** 1 Department of Health Administration and Policy, George Mason University, Fairfax, VA 22030, USA; xcheng4@gmu.edu (X.C.); slin26@gmu.edu (S.-y.L.); 2 Department of Biostatistics, School of Medicine, Virginia Commonwealth University, Richmond, VA 23219, USA; liuj32@vcu.edu; 3 Center for Governance Studies, Beijing Normal University at Zhuhai, Zhuhai 519087, China; shiyongliu2006@gmail.com; 4 Department of Physics and Engineering, Slippery Rock University of Pennsylvania, Slippery Rock, PA 16057, USA; jun.zhang@sru.edu; 5 Department of Economics, School of Economics and Finance, Xi’an Jiaotong University, Xi’an 710049, China; niepeng2017@xjtu.edu.cn; 6 Department of Health Behavior and Policy, School of Medicine, Virginia Commonwealth University, Richmond, VA 23219, USA; bernard.fuemmeler@vcuhealth.org; 7 Global Health Institute, School of Public Health, Xi’an Jiaotong University Health Science Center, Xi’an 710049, China; youfawang@gmail.com

**Keywords:** physical activity, obesity, machine learning, disparity

## Abstract

Background: Obesity prevalence has become one of the most prominent issues in global public health. Physical activity has been recognized as a key player in the obesity epidemic. Objectives: The objectives of this study are to (1) examine the relationship between physical activity and weight status and (2) assess the performance and predictive power of a set of popular machine learning and traditional statistical methods. Methods: National Health and Nutrition Examination Survey (NHANES, 2003 to 2006) data were used. A total of 7162 participants met our inclusion criteria (3682 males and 3480 females), with average age ranging from 48.6 (normal weight) to 52.1 years old (overweight). Eleven classifying algorithms—including logistic regression, naïve Bayes, Radial Basis Function (RBF), local k-nearest neighbors (k-NN), classification via regression (CVR), random subspace, decision table, multiobjective evolutionary fuzzy classifier, random tree, J48, and multilayer perceptron—were implemented and evaluated, and they were compared with traditional logistic regression model estimates. Results: With physical activity and basic demographic status, of all methods analyzed, the random subspace classifier algorithm achieved the highest overall accuracy and area under the receiver operating characteristic (ROC) curve (AUC). The duration of vigorous-intensity activity in one week and the duration of moderate-intensity activity in one week were important attributes. In general, most algorithms showed similar performance. Logistic regression was middle-ranking in terms of overall accuracy, sensitivity, specificity, and AUC among all methods. Conclusions: Physical activity was an important factor in predicting weight status, with gender, age, and race/ethnicity being less but still essential factors associated with weight outcomes. Tailored intervention policies and programs should target the differences rooted in these demographic factors to curb the increase in the prevalence of obesity and reduce disparities among sub-demographic populations.

## 1. Introduction

Over the last decade, the rapid rise in obesity prevalence has become one of the most prominent issues in global public health [1,2]. In 2015–2016, 39.6% of U.S. adults aged 20 and older and 18.5% of children and adolescents aged 2–19 were obese [3]. Obesity is of public health interest because excess fat would result in serious health consequences [4], including a high risk of hypertension, hypercholesterolemia, Type 2 diabetes [5], certain cancers [6], and early mortality [4]. As a result, the U.S. spends USD 315.8 billion annually treating obesity-related illness [7], and obesity is one of the top preventable causes of death in the U.S. [8].

Physical activity has been recognized as a key player in the obesity epidemic. It was found that the adherence rate to the physical activity (PA) guideline among U.S. adults remained low and unchanged between 2007 and 2016 [9]. Nationally representative data showed that in 2015, only 18% of the obese population met the PA guideline [10]. Previous evidence has suggested that a small increase in daily moderate-to-vigorous physical activity (5–10 min) was associated with a lower risk of obesity [11]. It was shown that those who maintained a healthy weight were consistently more likely to engage in vigorous physical activity [12]. Additionally, physical activity interventions can benefit children and adolescents with obesity [13] and reduce obesity-related health risks [14]. However, there are also other studies suggesting a low or null correlation between PA and weight status. For example, Lauran and others, using the Behavioral Risk Factor Surveillance System (BRFSS) and the National Health and Nutrition Examination Survey (NHANES), found that from 2001 to 2009, for every 1 percentage point increase in PA prevalence, obesity prevalence was 0.11 percentage points lower after controlling social detriments of health such as poverty, unemployment, and urbanicity [15]. Using the NHANES dated from 1988 to 2006, Ruth and colleagues revealed factors other than PA could contribute more to the increase in Body mass index (BMI) over time [16]. A prior systematic review concluded that, even though the health benefit of PA has been well documented, PA alone might be a minor determinant of obesity [17,18].

The relationship between physical activity and obesity remains unknown, given the mixed evidence in the extant literature. Thanks to technological advancement in recent years, machine learning (ML) has become an available powerful tool to help us identify the complex risk factors of the obesity problem. ML provides a novel way to analyze multifactorial data that can be further used to make predictions about the complex inter-relationships that likely drive the risk for obesity. Comparing with statistical modeling, machine learning learns from data without relying on rules and by not only focusing on relationships between variables in a way to circumvent issues regarding overfitting, collinearity, and assumptions that are crucial in regression models. Machine learning can handle extremely large volumes of highly complicated big data much better than statistical modeling [19,20]. The ML techniques, however, have not always performed better in clinical settings. A systematic review showed no performance benefit of ML models over traditional logistic regression for clinical prediction models [21]. In addition, there are some hurdles existing in ML-based prediction models that undermine the usefulness of this method. For instance, a better performing ML model, in terms of some accuracy measurements, will often be in conflict with the human understanding of predictions, and there is no rule of thumb for how much performance improvement is sufficient to justify using less interpretable estimators [22]. As a result, in the present exploratory study, we aimed to: (1) examine the relationship between PA and weight status using ML techniques and objectively measured national PA data and (2) compare the findings and performance of using ML-based and traditional statistical technique-based methods in assessing the relationship between PA and weight status. We hypothesized that PA is an important predictor of whether one encounters obesity or not and that ML models could yield a better predicting power in comparison with traditional statistical methods.

## 2. Materials and Methods

### 2.1. Data

The data used were from the National Health and Nutrition Examination Survey (NHANES) [23] 2003–2004 and 2005–2006. The NHANES was conducted by the Centers for Disease Control and Prevention (CDC). It is a nationally representative cross-sectional survey that aims to investigate the health and nutrition statuses of Americans [23]. PA data collected by a physical activity monitor (PAM) contain objective information on the intensity and duration of common locomotion activities. Respondents wore ActiGraph AM-7164 devices [24] for seven consecutive days. The device was placed on a flexible fabric belt that fitted each individual and was worn on the right hip [25]. The advantage of using objective measurement is to reduce the recall and reporting bias commonly seen in self-reported surveys. The accelerometer in ActiGraph AM-7164 recorded the sum of the magnitude of acceleration during every one-minute epoch. The sum of device intensity values (the magnitude of acceleration) and the duration (the sum of minutes) of PAs of different intensity during the seven days were both used in the experiments.

The data were collected through both interviews and physical examinations. In this study, we used the NHANES 2003–2004 (n = 10,122) and 2005–2006 (n = 10,348) mainly because the PAM was first available in these two waves (2003–2004 and 2005–2006). The analytic sample was restricted to adults aged 20–85 for whom detailed demographic, PA, and anthropometric information (including weight and height) was available for two waves. In addition, we excluded female respondents who were pregnant at the time of the survey. The final analytical sample totaled 7162.

### 2.2. Outcome Variables

BMI and overweight or obese weight status are the primary outcomes of this study. BMI was calculated as the respondent’s weight (kg) divided by squared height (m^2^). We adopted the clinical definition of overweight and obesity from 2000 CDC growth charts—normal weight is defined as 18.5 ≤ BMI < 25 kg/m^2^, overweight as 25 ≤ BMI < 30 kg/m^2^, and obesity as BMI ≥ 30 kg/m^2^ [26].

### 2.3. Exposure Variables

PA levels were grouped into five categories: sedentary (intensity < 100 counts/min), light (100 ≤ intensity < 760 counts/min), lifestyle (760 ≤ intensity < 2200 counts/min), moderate (2200 ≤ intensity < 6000 counts/min), and vigorous (intensity ≥ 6000 counts/min) [27].

### 2.4. Covariates

Covariates in the analyses were sociodemographic variables including gender, age, race, education level, marital status, and family poverty income ratio (PIR). Specifically, race was measured through 5 racial categories: non-Hispanic white, non-Hispanic black, Mexican American, other Hispanic, and other race (including multi-racial). The education level was the highest grade or level of school the individual had completed or the highest degree the individual had received. Marital status was measured on a 6-point scale: married, widowed, divorced, separated, never married, and living with partner. The family poverty income ratio (PIR) was defined as the ratio of the individual’s family income to the national poverty threshold.

### 2.5. Machine Learning and Statistical Analyses

This present study used eleven classification algorithms and traditional logistic regression to examine the relationship between PA and weight status. The eleven classification algorithms were naïve Bayes, radial basis function (RBF), local k-nearest neighbors (KNN), classification via regression (CVR), random subspace, decision table, multiobjective evolutionary fuzzy classifier, random tree, J48, and multilayer perceptron classification [28,29]. The naïve Bayes classifier is based on Bayes’ theorem [30]. The conditional distribution is learned under the assumption that all attributes are mutually independent. This technique has low complexity and high scalability. However, if the independence assumption is unmet, it may not perform well. The RBF classifier [31] is a supervised learning method. It uses Gaussian radial basis function networks and normalizes all attributes. The local KNN classifier detects k-nearest neighbors of one object in the attributes space with local metric induction [32,33]. The class to which the object is assigned rests on the classes to which its nearest neighbors belong. Although the KNN classifier is relatively easy to understand and implement, its primary disadvantage is that it would not perform well when attributes are heterogeneous and data are imbalanced. The CVR classifier uses regression models to evaluate the class value [31]. However, the class value should be binary. Both the random subspace and J48 classifiers [34] are based on tree-like models. One attribute is tested in one node. The random subspace classifier is based on decision trees, constructing trees in randomly chosen subspaces [35]. A decision table classifier builds a decision table and makes decisions by following the rules in the table [36]. Splitting one decision table into smaller ones is relatively simple but scaling up is more difficult. Although decision trees are well suited for classification of obesity, their greedy algorithm generates an approximation of the optimal decision tree and therefore may be more computationally complex and complicated to implement [37]. The multiobjective evolutionary fuzzy classifier is a fuzzy, rule-based model to optimize two objectives [38,39,40]. It generates comprehensible fuzzy rules to obtain optimal solutions by maximizing the accuracy and minimizing the number of fuzzy rules, but generating these rules is time-consuming [38]. Finally, the multilayer perceptron classifier is a kind of feedforward neural network [38] with high accuracy, but the computational complexity is high and requires a large amount of data. It utilizes a backpropagation-supervised learning technique for training [41,42]. The major advantage of modeling using neural networks is that they are self-adaptive models and are capable of approximating any functional form as closely as desired [43].

### 2.6. Terminology of Model Evaluation Tools and Definition

To evaluate the predictability of eleven algorithms, we used overall accuracy, sensitivity, specificity, and the receiver operating characteristic (ROC) curve. Overall accuracy is defined as the proportion of samples that are predicted correctly. Sensitivity, known as the true positive rate, is defined as the proportion of actual positives that are correctly detected. In our study, prediction of overweight or obesity was considered as the positive. It measured the probability of the classifier to accurately detect the individuals who were at risk of being overweight (or obese). Specificity is the true negative rate, indicating the proportion of actual negative (non-overweight or non-obese) individuals who are identified by the classifiers as negative. Additionally, normal weight was considered to be the negative. The receiver operating characteristic (ROC) curve is a useful tool for organizing classifiers and visualizing their performance. It is a curve located in a two-dimensional plot where the x axis is the false positive rate and the y axis is the true positive rate. The area under the curve (AUC) value can be calculated from the ROC curve. A higher AUC value indicates a better model performance.

To evaluate the importance of each predictor, we used an information gain algorithm to rank the contribution of each predictor. Information gain is based on the concept of reduction in entropy. Entropy, in information theory, measures the amount of information that is missing before reception. It is a concave function with 0 being the smallest value and 1 being the largest in binary learning tasks and thus suitable for the present study [36]. A factor with high information gain was ranked higher because it has stronger classifying power. All analyses were conducted using WEKA or SAS.

## 3. Results

Table 1 reports the key characteristics of the study sample, including demographic, anthropometric, and physical activity features. There were 7162 participants in the sample set, 51.41% of whom were male. The individuals in the study consisted of adults from 20 to 85 years old. The mean age of the obese group (50.02) was lower than the overweight group (52.13) and higher than the healthy group (48.60). There were five racial categories: non-Hispanic white, non-Hispanic black, Mexican American, other Hispanic, and other race (including multi-racial). The education level was the highest grade or level of school the individual had completed or the highest degree the individual had received. There were six types of marital status: married, widowed, divorced, separated, never married, and living with partner. Only 117 individuals refused to answer this question. The family poverty income ratio (PIR) is the ratio of the individual’s family income to the national poverty threshold. The lowest value is 0, and the highest value is 5. The mean family PIR of the overweight class was 2.74, which was higher than the other two groups. The mean value of the sum of intensity values had a positive association with group BMI. The sum of intensity values of obese groups was lower than the other two groups. The obese group also had the longest duration of sedentary activities and the shortest duration of other types of activities. For physical activity variables, the difference between the healthy and overweight groups was generally not obvious.

### 3.1. Relationship between PA and Overweight/Obesity

We first predicted whether an individual was overweight/obese based on PA levels. The individuals were divided into two groups based on their BMIs: normal and abnormal (including both overweight and obese individuals). Table 2 represents the classification performance results. The random subspace classifier had the highest overall accuracy of 70.01%. The sensitivity of the J48 classifier was 72.9%. The highest specificity was 57.3% for the random subspace classifier, while the lowest specificity was 35.6% for the random tree model. The mean percentages of overall accuracy, sensitivity, and specificity of the eleven classifiers were 62.37%, 70.89%, and 49.70%, respectively. The sensitivities for all models were much higher than the corresponding specificities.

**Figure 1:**
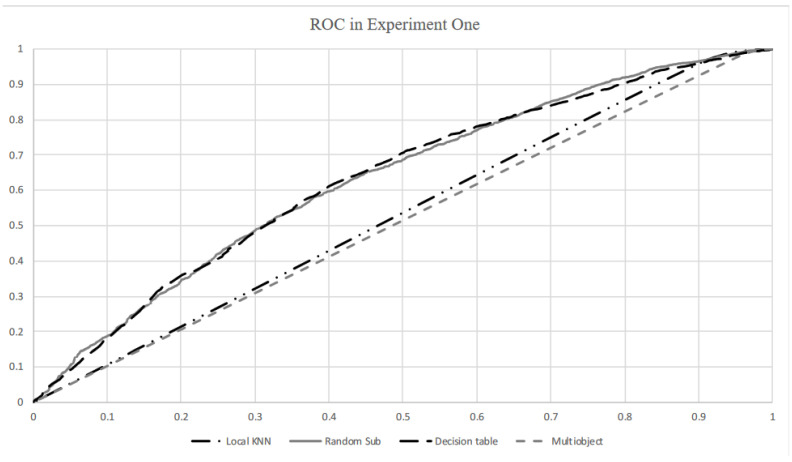
Receiver operating characteristic (ROC) curves for local KNN, random subspace, decision table, and multiobject algorithms.

The sensitivity of the logistic regression method was slightly lower than the mean value, while the specificity was slightly higher than the mean value. The random tree performed worst in overall accuracy and specificity, although it achieved a higher sensitivity compared with the mean sensitivity value. In general, all the methods have similar overall accuracy, sensitivity, and specificity. As shown in the ROC curves in Figure 1, the random subspace classifier produced the highest result with 63.3% area under the ROC curve (AUC). The other four classifiers, namely, RBF, CVR, decision table, and multilayer perceptron, also achieved marginally over 62% AUC. Naïve Bayes and J48 classifiers proved average in AUC, with approximately 60%. The AUC values of local KNN and random tree classifiers were slightly higher than 53%. The AUC value from the multiobject classifier was the lowest of 51.2%.

### 3.2. Relationship between PA and Obesity Only

We then predicted whether an individual was obese based on PA levels. The individuals were labeled as not obese and obese. The results of classifying are shown in Table 3. The mean overall accuracy was 57.69%, which was lower than that of Table 2 (62.37%). Likewise, the mean sensitivity was 48.55%, which was much lower than that of Table 2 (70.89%). Nonetheless, the mean specificity was 68.75%, which was significantly higher than that of Table 2 (49.70%). The random subspace classifier had the highest overall accuracy and sensitivity. Compared with other methods, naïve Bayes had the highest specificity (76.7%). However, the overall accuracy (49.64%) of the naïve Bayes classifier was significantly lower than that of the other ten classifiers. Regarding overall accuracy, the logistic regression model performed slightly worse than the other algorithms, excluding naïve Bayes, random tree, and J48, all of which performed lower than the logistic regression classifier. Both the sensitivity (49.4%) and specificity (66.5%) of logistic regression were slightly higher than the mean values of all eleven models. Table 3 also demonstrates that for all of the classifiers, specificities were higher than sensitivities. The sensitivities of six algorithms (including logistic regression, naïve Bayes, multiobject, random tree, J48, multilayer perceptron) were lower than 50%. The highest sensitivity obtained from random subspace was only 56.8%.

**Figure 2:**
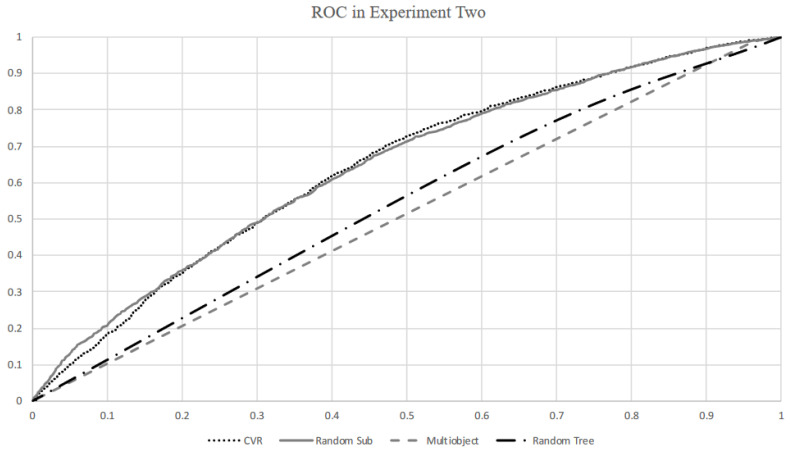
ROC curves for CVR, random subspace, random tree, and multiobject algorithms.

Figure 2 illustrates the ROC curves of the four classifiers that achieved the two highest AUC values and the two lowest AUC values. Both random subspace and CVR classifiers achieved 64.3% AUC values. However, RBF, decision table, and multilayer perceptron classifiers performed slightly worse than the random subspace classifier. The naïve Bayes classifier achieved 62% AUC in obesity prediction. The multiobject classifier still had the lowest AUC of 51%. In general, all classifiers produced similar AUC values to those of Table 2.

### 3.3. Importance of PA in Predicting Weight Status

We further checked variable importance by ranking all features involved in predictive models. All features were ranked by an information gain ranking filter. One Rule is a classifier that only generates one rule for each feature. The importance of a feature was evaluated by the accuracy of the rule. Table 4 shows the feature ranking results.

Duration of vigorous-intensity activity in one week and duration of moderate-intensity activity in one week were ranked top among 12 features with contributions of 0.0211 and 0.014, respectively. Age was ranked No.3 with a 0.0137 contribution. Gender, race/ethnicity, and socioeconomic status (SES) (marital status, education level, and poverty income ratio), even though they contributed to the prediction model, had less predictive power, ranking among the bottom.

## 4. Discussion

This study is the first study in the field to assess adulthood overweight/obese risks using objectively measured PA data and advanced ML techniques. Our results indicate that PA (especially moderate to vigorous intensity) was a key risk factor in predicting overweight and obesity. In addition, duration of moderate-intensity activity and duration of vigorous-intensity activity ranked higher than the sum of the intensity value recorded by the physical activity monitor, duration of sedentary-intensity activity, duration of lifestyle-intensity activity, and duration of light-intensity activity. The results imply that high-intensity physical activity is more important than low-intensity physical activity and overall physical activity intensity. This finding aligns with our hypothesis and empirical evidence. Physical activity increases a person’s energy expenditure and helps individuals maintain their energy balance or even lose weight as long as energy consumption is not compensated by calorie intake [44]. Demographic features played a less but still critical role in predicting weight status as our ML and traditional statistical analyses suggested. This finding is in line with previous studies [15,45]. Underlying disparities associated with race/ethnicity including food environment, healthcare access, social environment, and building environment are important factors that predict the risk of obesity through complex known and unknown pathways [46]. This may provide guidance for obesity control.

We compared the performance of different ML algorithms in predicting weight status and found that the random subspace algorithm had a weak superiority over the other ten models. However, the logistic regression model performed slightly worse than the random subspace and several other algorithms. The random subspace classifier produced the highest overall accuracy and AUC among the eleven classifiers. It also produced the highest specificity for prediction of normal weight and the highest sensitivity for prediction of obesity. The sensitivity of the J48 classifier in normal weight prediction was 2.3% higher than the sensitivity of the random subspace classifier. The specificities of the J48 classifier in both normal weight and obesity predictions were also higher than the random subspace classifier. The overall accuracies of the J48 classifier were lower than those of the random subspace classifier. Furthermore, its sensitivity was 11.0% lower than the sensitivity of the random subspace classifier in prediction of obesity. Overall, the random subspace classifier outperformed the J48 classifier. Regarding obesity prediction, the specificity produced by the naïve Bayes classifier was 8.7% higher than that of the random subspace classifier. However, it performed worse than the other algorithms regarding overall accuracy and sensitivity.

Our results are comparable to existing studies. For instance, Jie et al. [47] reviewed 927 studies and provided no evidence of the superior performance of advanced machine learning methods over the logistic regression method for clinical predictions. Similarly, Tozlu et al. [48] used logistic regression and four other machine learning methods (adaptive boosting, ANN, RF, and SVM) to identify high infarction risk. They concluded that logistic regression performed as well as the other four machine learning approaches. In addition, Tozlu et al. [48] compared the performances of several machine learning models to predict the effect of labor induction on the occurrence of cesarean section and found that the logistic regression model produced a similar performance to the RF one. Therefore, advanced machine learning methods with high computational complexity are not always necessary in obesity prediction. Due to the well-understood theoretical and computational background, the logistic regression model is preferable when predicting obesity.

Some limitations of this study should be noted: First, ActiGraph AM-7164 is not waterproof, and thus some activities such as swimming were not captured. Thus, the collected data for some individuals who performed aquatic activities are not trustable. The device may not have been able to accurately detect upper-body exercises, such as lifting dumbbells. Second, this study only examined weight status but not body composition, which is planned for our future research.

## 5. Conclusions

This study was conducted in a large population-based sample and thus the results reflect the average relationship between physical activity and BMI. Weight loss intervention trials testing the addition of physical activity to diet modification have shown the importance of physical activity at weight-loss maintenance. The findings herein do not speak to the types and level of physical activity needed for individuals trying to lose weight.

In sum, the random subspace algorithm had a weak superiority over the other ten models including logistic regression. Although physical activity was a crucial predictor of weight outcomes, demographic characteristics including gender, age, and race/ethnicity were also important factors associated with weight outcomes. Tailored intervention policies and programs should target the differences rooted in these demographic factors to curb the increase in the prevalence of obesity and reduce disparities among sub-demographic populations.

## Figures and Tables

**Table 1:** Study population characteristics.

Variables	Normal (18.5 ≤ BMI < 25 kg/m^2^)		Overweight (25 ≤ BMI < 30 kg/m^2^)		Obese (BMI ≥ 30 kg/m^2^)	
	Observations	Mean (%)	Observations	Mean (%)	Observations	Mean (%)
Gender						
Male	1046	47.72	1505	59.65	1131	46.22
Female	1146	52.28	1018	40.35	1316	53.78
Age	2192	48.60	2523	52.13	2447	50.02
Race						
Non-Hispanic White	806	36.77	857	33.97	708	28.93
Non-Hispanic Black	334	15.24	368	14.59	508	20.76
Mexican American	420	19.16	593	23.50	606	24.77
Other Race, Including Multi-Racial	342	15.60	396	15.70	410	16.76
Other Hispanic	290	13.23	309	12.25	215	8.79
Education Level						
Less than 9th Grade	702	32.03	917	36.35	862	35.23
9–11th Grade (Includes 12th Grade with No Diploma)	251	11.45	280	11.1	292	11.93
High School Grad/GED or Equivalent	347	15.83	438	17.36	465	19
Some College or AA Degree	344	15.69	369	14.63	400	16.35
College Graduate or Above	451	20.57	421	16.69	341	13.94
Refused	95	4.33	95	3.77	87	3.56
Do Not Know	1	0.05	1	0.04	0	0
Marital Status						
Married	807	36.82	1016	40.27	903	36.90
Widowed	445	20.30	514	20.37	485	19.82
Divorced	305	13.91	331	13.12	356	14.55
Separated	148	6.75	203	8.05	219	8.95
Never Married	327	14.92	276	10.94	319	13.04
Living with Partner	126	5.75	143	5.67	122	4.99
Refused	34	1.55	40	1.59	43	1.76
Family PIR	2192	2.62	2523	2.74	2447	2.60
Sum Intensity Value	2192	1,584,527.52	2523	1,562,816.99	2447	1,298,389.77
Duration of Different Activity Intensity Levels (in Minutes)						
Sedentary	2192	7988.89	2523	7945.10	2447	8146.58
Light	2192	1468.27	2523	1498.67	2447	1407.09
Lifestyle	2192	500.84	2523	522.37	2447	450.55
Moderate	2192	112.70	2523	106.91	2447	73.21
Vigorous	2192	7.45	2523	4.63	2447	1.93

**Table 2:** Evaluations of accuracy, sensitivity, and specificity of 11 prediction models of overweightness and obesity.

Method	Logistic Regression	Naïve Bayes	RBF ^†^	Local KNN ^†^	CVR ^†^	Random Subspace	Decision Table	Multiobject	Random Tree	J48 ^‡^	Multilayer Perceptron	Mean Value of 11 Models
Accuracy	69.4%	69.11%	69.5%	69.63%	69.9%	70.01%	69.59%	69.37%	60.71%	68.70%	68.65%	62.37%
Sensitivity	70.0%	70.8%	70.2%	70.7%	70.9%	70.6%	70.1%	70.0%	71.6%	72.9%	72.0%	70.89%
Specificity	51.6%	48.1%	51.6%	52.1%	54.4%	57.3%	51.4%	49.5%	35.6%	47.9%	47.2%	49.70%

Notes: ^†^ RBF = radial basis function; KNN = local k-nearest neighbors; CVR = classification via regression; random subspace; ^‡^ J48 = J48 is an algorithm to generate decision trees.

**Table 3:** Evaluations of accuracy, sensitivity, and specificity of 11 prediction models of obesity.

Method	Logistic Regression	Naïve Bayes	RBF ^†^	Local KNN	CVR	Random Subspace	Decision Table	Multiobject	Random Tree	J48 ^‡^	Multilayer Perceptron	Mean Value of 11 Models
Accuracy	65.78%	49.64%	66.20%	65.92%	65.89%	67.03%	66.32%	65.78%	58.42%	63.66%	64.48%	57.69%
Sensitivity	49.4%	38.6%	52.7%	50.3%	50.3%	56.8%	52.4%	49.1%	39.5%	45.8%	49.2%	48.55%
Specificity	66.5%	76.7%	67.1%	68.1%	67.2%	68.0%	67.9%	66.3%	68.7%	69.9%	69.9%	68.75%

Notes: ^†^ RBF = radial basis function; KNN = local k-nearest neighbors; CVR = classification via regression; random subspace; ^‡^ J48 = J48 is an algorithm to generate decision trees.

**Table 4:** Ranked feature importance in predicting weight status based on information gain.

Rank	Feature Meaning	Contribution
1	Duration of moderate-intensity activity in one week	0.0211
2	Duration of vigorous-intensity activity in one week	0.0140
3	Age	0.0137
4	The sum of the intensity value recorded by the physical activity monitor in one week	0.0133
5	Race/Ethnicity	0.0110
6	Duration of sedentary-intensity activity in one week	0.0053
7	Duration of lifestyle-intensity activity in one week	0.0045
8	Duration of light-intensity activity in one week	0.0042
9	Gender	0.0040
10	Education level	0.0027
11	Poverty income ratio (PIR)	0
12	Marital status	0

## Data Availability

Data sharing not applicable.
